# Bi-planar calibration method for templating of hip joint arthroplasty: phantom study and proof of concept

**DOI:** 10.1007/s00264-023-05747-4

**Published:** 2023-03-07

**Authors:** Christoph Kolja Boese, Tim Rolvien, Frank Oliver Henes, Frank Timo Beil, André Strahl, Christian Ries

**Affiliations:** 1 Department of Trauma and Orthopaedic Surgery, Division of Orthopaedics, University Medical Center Hamburg-Eppendorf, Martinistr. 52, D-20246 Hamburg, Germany; 2 Center for Radiology and Endoscopy, Department of Diagnostic and Interventional Radiology and Nuclear Medicine, University Medical Center Hamburg-Eppendorf, Martinistr. 52, D-20246 Hamburg, Germany

**Keywords:** Digital templating, Total hip arthroplasty, Joint arthroplasty, Arthroplasty, Digital radiography

## Abstract

**Purpose:**

Calibration of radiographs is a critical step in digital templating for hip arthroplasty. Calibration errors of > 1.5% lead to over- or undersizing of the templated implants and may affect logistics and patient safety. Contemporary calibration methods are known to be imprecise with average errors of 6.5% and wide variance. A novel bi-planar radiograph-based calibration method is proposed, and a phantom study was conducted as proof of concept.

**Methods:**

A spherical external calibration marker (ECM) is placed in front of the pubic symphysis of a pelvic bone model at twelve different positions. For each marker position, standard anteroposterior radiographs and four corresponding lateral radiographs with different degrees of rotation (0°–30°) are taken (overall, 60 radiographs). Calibration factors are calculated for an internal calibration marker (ICM) at the centre of the right hip (reference) and the ECM using a novel algorithm. Rotation and marker positions simulate foreseeable use errors and misplacements and aim to test robustness of the method against these errors.

**Results:**

ECM calibration factor was 125.9% (range 124.7–127.2), and the mean ICM calibration factor was 126.6% (range 126.2–127.1) ($p<0.001$). Four images (8.3%) were beyond the 1% error threshold (all with 30° rotation). The mean difference was 0.79% (SD 0.49).

**Conclusion:**

The bi-planar method precisely predicts the true calibration factor of the hip joint plane under various conditions. In lateral radiographs, rotation of up to 20° did not adversely affect the precision and all images had calibration errors below the threshold for clinical significance.

**Supplementary Information:**

The online version contains supplementary material available at 10.1007/s00264-023-05747-4.

## Introduction

Digital templating on radiographs is a standard method prior to joint arthroplasty and an integral part of quality and risk management [1]. A key step in digital templating is calibration of the digital radiograph to the plane of interest (i.e., the hip plane in total hip arthroplasty) which is required to adequately template implant size and individual reconstruction of anatomical aspects of the hip joint [1, 2]. Conventionally, the focus of templating is the assessment and recognition of individual anatomy and biomechanical parameters to foster adequate implant selection and reconstruction. Here, a precision of ± 1 implant size has been widely accepted as sufficient [3]. However, current calibration methods have been shown to be inadequate and the proposed precision has only been achieved in about 74% of uncemented stems and 73% of uncemented cups [3]. In addition, more recent developments in digitalization aim to improve process flows to integrate templating into patient and device logistics from plan to procedure. Reliable integration of templating into surgery with computer-assisted surgery and robotics may require a precision beyond ± 1 implant size. Therefore, novel methods are needed.

Usually, external calibration markers (ECM) are placed in defined positions [1, 4, 5]. However, established methods are associated with a significant lack of precision and reliability [2, 4, 6, 7]. More recently, improvements of calibration methods were based on predictive models with dual-scale markers in combination with empirical data [8–10]. Still, the precision is below expectations and further improvements are required [11, 12]. Alternatively, 3D templating with tomography imaging is possible [13]. However, CT-based imaging is associated with increased costs and radiation exposure to the patient. MRI-based imaging is time-consuming and not regularly available to the patient nor cost-effective [1].

The calibration factor (also known as magnification factor) of radiographs is usually reported as percent magnification (%). Errors of calibration markers are reported as relative or absolute difference from the true magnification factor of the target plane (e.g., hip plane). These differences are also reported as percent. It is known that calibration errors of about 2–3% will result in deviation of approximately one component size and about 2 mm of error on measurement of lengths [12]. More precisely, the error increases with size of the implants. For commonly used hip implants, an error of ≤ 1.5% is acceptable as it should not result in deviations from optimal implant size selection [12]. This can be considered the minimal clinically important difference (MCID). While this threshold is more ambitious that the traditional view of ± 1 implant size, it reflects the goal to further improve templating in general and allow for future developments of integrated computer-assisted surgery (e.g., robotic surgery).

In conventional templating, the average error has been shown to be approximately 6.5% with errors of up to 28.6% [2, 4, 14]. More recently, dual-scale methods have shown mean errors of 2.1% (up to 9.5%) in standing and 1.1% (up to 5.6%) in supine radiographs [9, 10]. The study in supine subjects is limited as standing radiographs are usually the standard in pre-operative templating. In supine radiographs, only 55% were below the reported threshold of 1% error [9]. In standing radiographs, the error of ≤ 1% was found in 35% of the cases and an error of ≤ 1.5% in 47% of the cases, leaving more than half of the cases beyond a clinically relevant cut-off [10].

To improve validity and reliability of templating and thereby reducing the frequency of calibration errors, a novel method has been proposed relying on bi-planar radiographs. Here, the true position of the calibration marker and the hip plane is calculated without need for empirical data and prediction models. A marker is attached to the patient in front of the pubic symphysis, and a lateral radiograph of the hip is taken in addition to the anteroposterior pelvic radiograph for templating. Following a proprietary algorithm, the magnification of the hip plane is calculated.

The method does not require special equipment and could be implemented in any facility performing radiographs for pre-operative planning. The only requirements are (1) a standard marker ball attached to the patient during two approximately orthogonal radiographs and (2) special software to perform the measurements and calculations.

The primary aim of the study is the proof of concept of the bi-planar calibration method in a phantom study. Secondary research objectives are (1) the quantification of the precision of the method, (2) assessment of the reliability of the method, (3) identification of sources of error of the method, and (4) subgroup analysis for precision based on rotation of the phantom.

## Materials and methods

An X-ray phantom was created to allow positioning of an external calibration marker (ECM) in various positions relative to the (right) hip centre and thereby the hip plane as target plane for templating. The hip centre was represented by an internal calibration marker (ICM). The ECM could be positioned in various positions representing clinically relevant position errors away from the optimal position in the midline before the pubic symphysis. The pelvic model was added to demonstrate anatomical conditions. In addition, the model could be rotated to defined positions to address potential errors of patient positioning during the lateral radiographs.

### Phantom

**Fig. 1:**
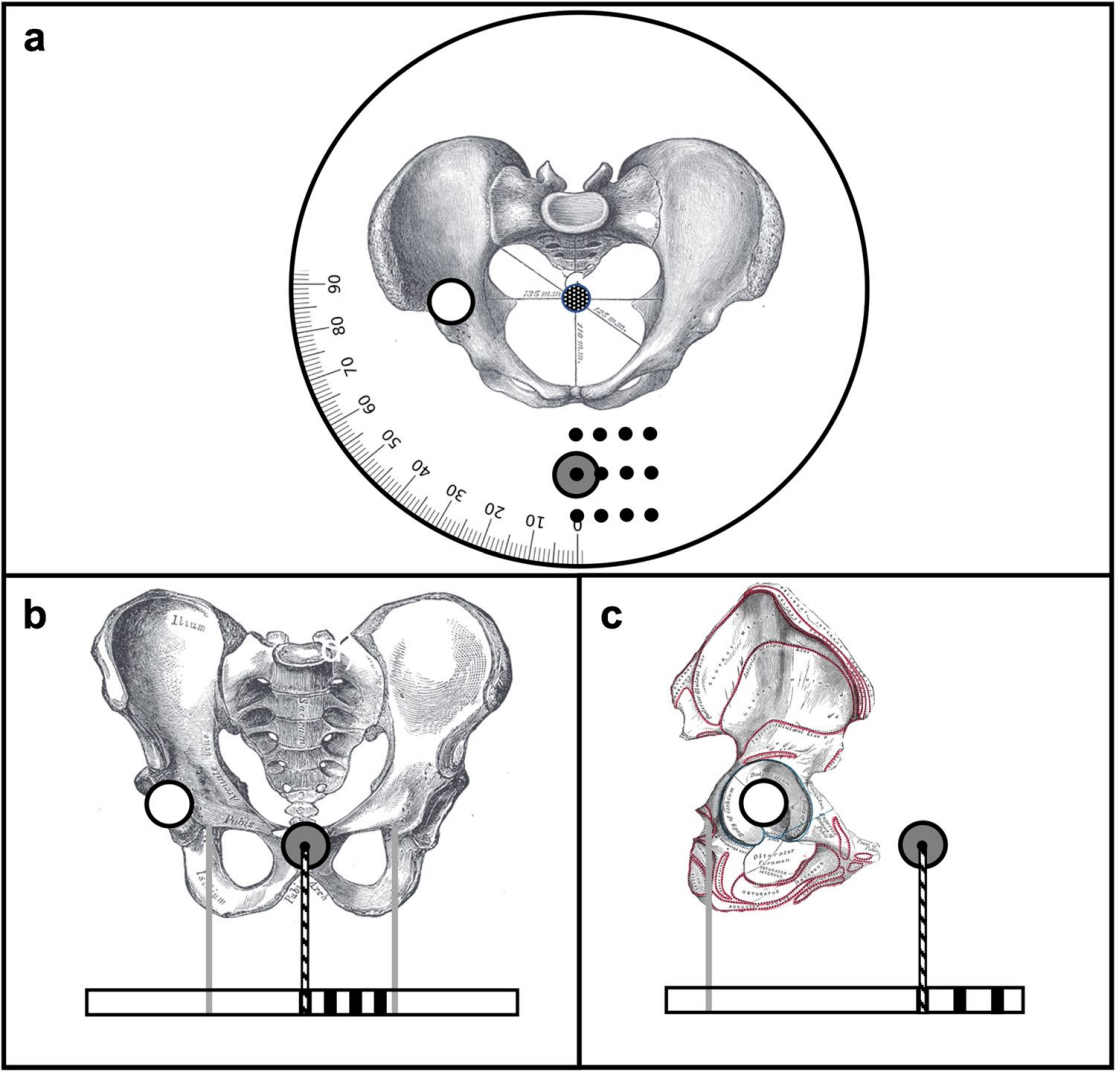
X-ray phantom setup in three planes. ECM = black circle with gray fill; ICM = black circle with white fill; variable ECM positions = small black filled circle. **a** Top view. **b** Coronal view. **c** Lateral view. Pelvis sketches adapted from *Henry Gray (1918) “Anatomy of the Human Body”* in public domain (Figures 238 (a), 241 b and, 235 (c))

A model of the pelvis (Sawbones, USA, Pelvis, Full Male, Solid Foam, SKU: 1301) was fixed to a stand. The stand allowed for 360° rotation of the model with an indicator and scale of rotation. The model was positioned with about 10° pelvic tilt and hence a natural alignment of the anterior pelvic plane. The setup of the model is shown in Fig. 1.

#### Internal calibration marker (ICM)

A radiopaque metal sphere (cobalt-chromium femoral ball head, 32 mm diameter) was fixed to a wooden stand. It was positioned centrally in the acetabulum on the right side of the pelvic model. The marker was used as internal reference (ICM) for the bi-planar external calibration marker (ECM).

#### External calibration marker (ECM)

The external marker ball (stainless steel ball, 25 mm diameter) was fixed to a threaded rod to allow repositioning of the marker relative to the ICM and pelvic model.

#### Base plate and ECM settings

The base plate allowed fixation of the ECM rod in various positions relative to the pubic symphysis. Three positions (anterior offset; 20, 50, and 80 mm anterior to the pubic symphysis) could each be combined with four positions of lateral offset from the midline (0, 20, 40, or 60 mm). Therefore, twelve different positions of the ECM were possible.

The anterior offset simulated different patient sizes. Based on data from a series of 400 CT scans, the mean distance for soft tissue anterior to the pubic symphysis was 41 mm (14–117 mm, SD 16). Most cases were below 80 mm and only a small fraction was above.

The lateral offset simulated potential misplacement of the ECM. While it should be positioned in the central beam which in turn is centered to the pubic symphysis (in a.p. radiographs), deviation from the central beam in any direction has the same projectional effect. Misplacement of up to 60 mm lateral offset from the central beam was considered. Higher offset should be obvious during placement and be recognized before radiographs are taken.

#### Rotation

The phantom was rotated in defined positions of 0°, 10°, 20°, and 30° in the lateral radiograph setting. Therefore, one anteroposterior (a.p.) and four lateral radiographs were taken for each ECM setting resulting in overall twelve a.p. and 48 lateral radiographs.

Rotation of the phantom in lateral radiographs simulated deviations of the patient position from the strictly lateral position. A rotation of more than 30° seems clinically unlikely as it would be easily identified during patient positioning before the radiograph is taken.

### Radiography settings

For the anteroposterior radiograph, the central beam was centered to the pubic symphysis. For the lateral image, the central beam has been centered to the hip center and thus the center of the reference marker ball. A focus-detector distance of 1150 mm was applied in all radiographs.

#### Series and sets

In the following, a set was defined as one a.p. and one corresponding lateral radiograph. A series was defined as one a.p. radiograph with all four corresponding lateral radiographs. Overall, 12 series were created with 12 a.p. and 48 lateral radiographs (total of 60 radiographs).

### Radiograph analysis and measurements

All radiographs were stored in a picture archiving and communication system (PACS). Measurements were performed following a standardized protocol using a PACS client (IMPAX EE, Agfa, Mortsel, Belgium). All measurements were entered into a standardized spreadsheet.

After training in the method of image analysis and measurements, two observers performed the image analysis of all radiographs independently. For intrarater analysis, one observer analyzed randomly selected three series of five images, respectively (25%). This analysis was performed two to six weeks after the first measurements. The observer was blinded to the previous measurements.

#### Measurements

**Fig. 2:**
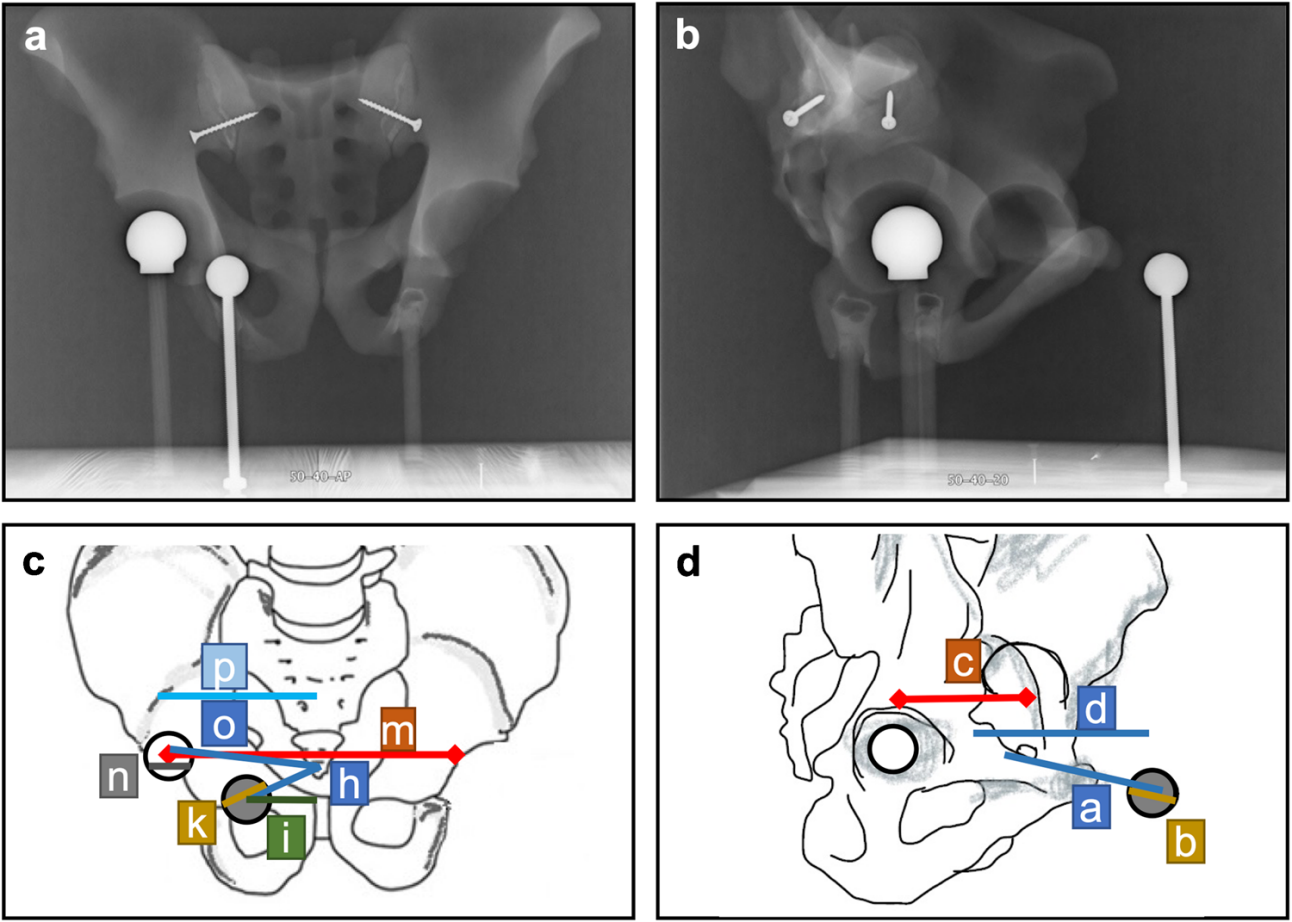
Depiction of measurements. A rotated lateral image (20°) is depicted to show all measurements. **a** a.p. radiograph and **b** lateral radiograph of the phantom. The ECM is positioned at 50 mm anterior and 40 mm lateral offset. **c** Sketch of a.p. radiographs with measurements of *h, i, k, m, n, o,* and *p*. **d** Sketch of lateral radiographs with measurements of *a, b, c,* and *d*. ECM = black circle with gray fill; ICM = black circle with white fill

Measurements included four distances in the lateral and seven distances in the a.p. radiograph. Measurements are listed in Table 1 and depicted in Fig. 2.

**Table 1:** Measurements

Variables of lateral radiograph *a* = direct distance image center to ECM center *b* = long half-axis ECM diameter *c* = horizontal distance right hip center to left hip center *d* = dist. horizontal hip center to ECM center
Variables of anteroposterior radiograph *h* = direct distance image center to ECM center *i* = horizontal distance image center to ECM center (i.e., lateral offset) *k* = long half-axis ECM diameter *m* = distance right hip center to left hip center *n* = long half-axis ICM diameter *o* = direct distance image center to ICM center *p* = horizontal distance image center to ICM center

### Calculation of calibration factors

Calibration factors were calculated following a proprietary algorithm. The reference calibration factor (ICM) was calculated using the method described by Boese et al. with multiple iterations [6, 10, 15]. The calibration factor of the ECM was calculated following the novel approach and algorithm to perform the bi-planar calibration method.

### Primary outcome and MCID

The relative and absolute differences of the ICM and ECM calibration factors were calculated for each radiograph set. A threshold of a difference of ≤ 1.5% magnification was considered acceptable. A difference of > 1.5% was considered unacceptable.

### Statistics

Statistical analysis was performed using a statistical program (SPSS, Windows, version 28.0, IBM, Chicago, IL). Continuous variables were expressed as mean, minimum and maximum, and standard deviation (SD). Box plots and histograms were generated.

#### Comparison of ICM and ECM calibration factors

T-tests were applied to investigate differences between the reference (ICM) and the ECM calibration factors. A $p$ value below 0.05 was considered statistically significant. Clinical relevance was defined as calibration factor difference of 1 (Supplemental Fig. S1). Subgroup analysis was performed with Kruskal–Wallis tests for each baseline setting (i.e., anterior offset, lateral offset, and rotation).

#### Reliability: intraclass correlation coefficient

Reliability of the measurements was assessed with intraclass correlation coefficients (ICC). Intrarater reliability was tested with the available repeated measurements of observer 1 in sixteen lateral and four a.p. radiographs. Interrater reliability was assessed for the two observers 1 and 2 in all radiographs ($n=48+12$). Outcomes are reported as supplemental material (Supplemental Tables T1 and T2).

### IRB

Due to the nature of the study, no IRB approval was required. No human subjects or animals were involved.

## Results

### Image analysis and dataset

**Fig. 3:**
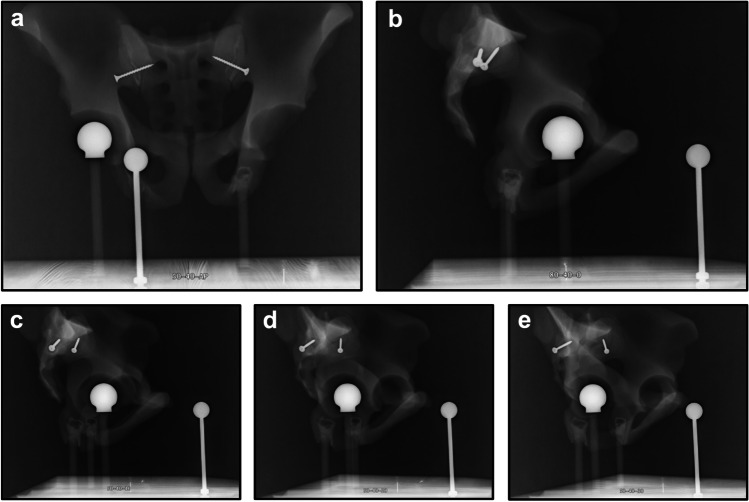
Depiction of a series of one a.p. radiograph with four corresponding lateral radiographs. In this example, the ECM is positioned at 50 mm anterior offset and 40 mm lateral offset. **a** a.p. radiograph and **b** lateral radiograph without rotation. **c** Lateral radiograph with 10° rotation. **d** Lateral radiograph with 20° rotation. **e** Lateral radiograph with 30° rotation

Overall, 60 radiographs, including twelve a.p. and 48 corresponding lateral radiographs, were included in the analysis (example of image set in Fig. 3).

### Primary outcome: precision of calibration

**Fig. 4:**
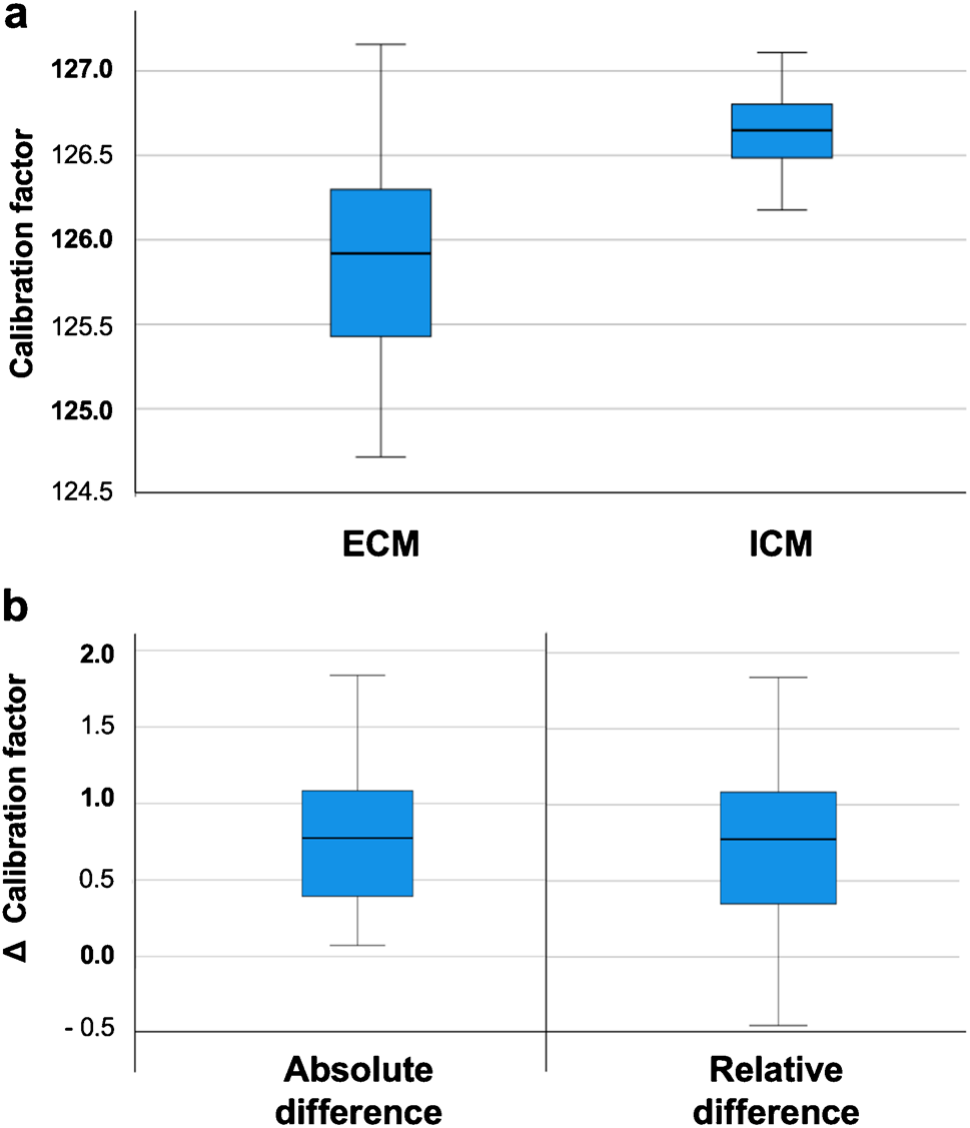
Box plots of **a** the ECM and ICM calibration factors and **b** absolute and relative differences of ECM and ICM calibration factors

The mean ECM calibration factor was 125.9% (range 124.7–127.2), and the mean ICM calibration factor was 126.6% (range 126.2–127.1). Therefore, the same calibration factor is the comparator for each series of bi-planar ECM calibration factors with four settings of rotation. Details are presented in Table 2. The relative and absolute differences are reported in Table 2, and box plots show the distribution of the ICM and ECM calibration factors as well as differences (Fig. 4).

**Table 2:** Descriptive statistics of ICM and ECM calibration factors and differences. Note: the same ICM calibration factor applies to all rotation settings for ECM calibration factors

		Calibration factor				
		Rotation				
		All	0°	10°	20°	30°
Mean	ICM	**126.65**	126.65			
	ECM	**125.91**	126.56	126.08	125.67	125.32
	Relative difference	**0.74**	0.09	0.57	0.97	1.33
	Absolute difference	**0.79**	0.30	0.57	0.97	1.33
Minimum	ICM	**126.18**	126.176			
	ECM	**124.71**	126.06	125.51	125.24	124.71
	Relative difference	** − 0.45**	− 0.45	0.08	0.47	0.78
	Absolute difference	**0.07**	0.07	0.08	0.47	0.78
Maximum	ICM	**127.11**	127.11			
	ECM	**127.16**	127.16	126.73	126.33	125.94
	Relative difference	**1.84**	0.66	1.00	1.51	1.84
	Absolute difference	**1.84**	0.66	1.00	1.51	1.84
Standard deviation	ICM	**0.24**	0.249			
	ECM	**0.57**	0.31	0.31	0.32	0.40
	Relative difference	**0.57**	0.36	0.30	0.30	0.38
	Absolute difference	**0.49**	0.18	0.30	0.30	0.38

A paired t-test showed significant differences ($p<0.001$) for ICM and ECM calibration factors. However, the mean absolute difference was 0.79% with a standard deviation of 0.49. The minimal clinically important difference (MCID) was set to be above 1.5.

**Fig. 5:**
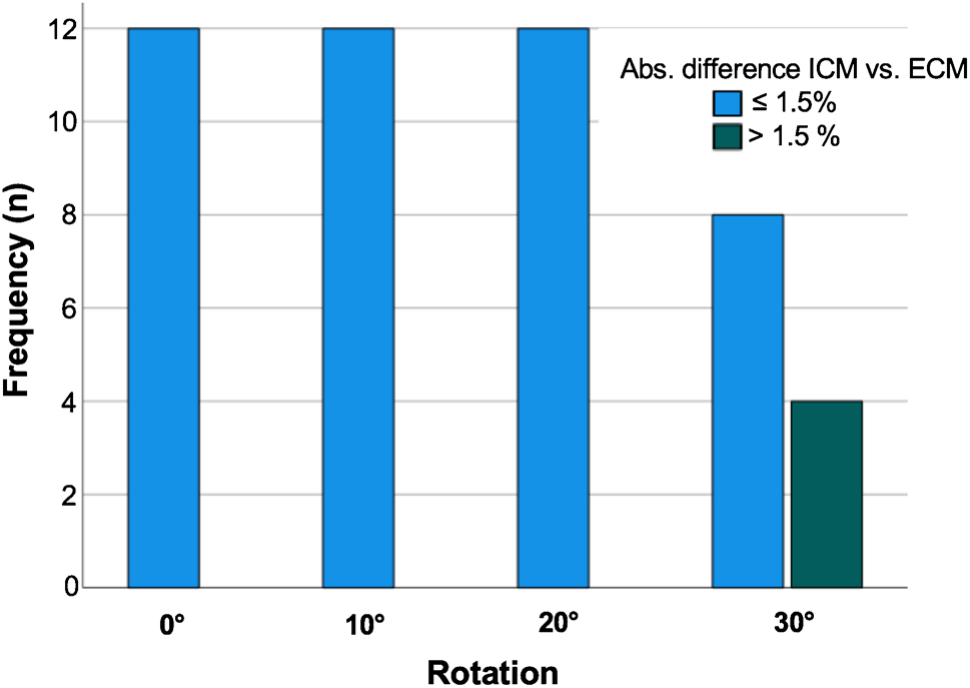
Histogram of frequency for radiographs with absolute differences within or above the minimal clinically important difference (MCID) of 1.5

The frequency of images with an absolute difference beyond 1.5% was four ($4/48 = 8.3\%$). Of note, all radiographs with differences beyond the MCID were in the group of 30° rotation (Fig. 5).

### Subgroup analysis

**Fig. 6:**
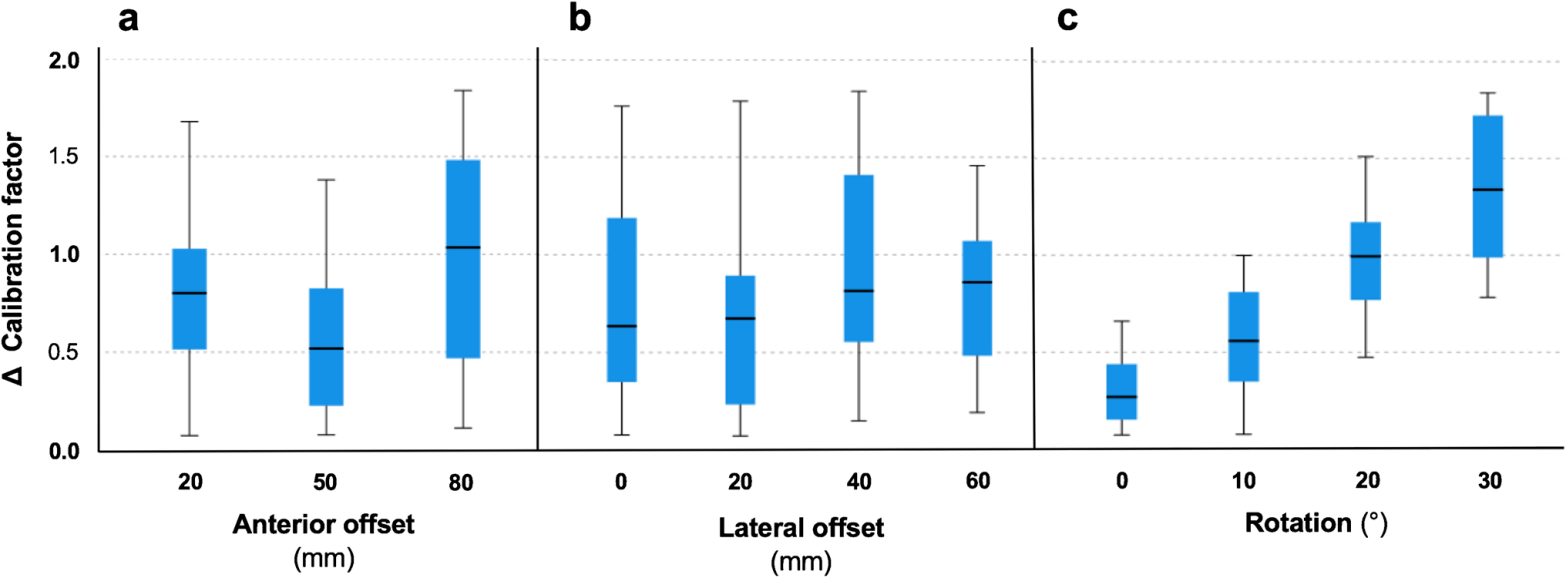
Box plots of absolute difference between ECM and ICM calibration factors. Plotted by **a** anterior offset, **b** lateral offset, and **c** rotation

Anterior offset did not significantly influence the absolute difference between ICM and ECM calibration factors ($p=0.067$, Kruskal–Wallis test, Fig. 6a). Lateral offset did not significantly influence the absolute difference between ICM and ECM calibration factors ($p=0.608$, Kruskal–Wallis test, Fig. 6b). In contrast to the aforementioned factors, rotation did significantly influence the absolute difference between ICM and ECM calibration factors ($p<0.001$, Kruskal–Wallis test, Fig. 6c).

**Fig. 7:**
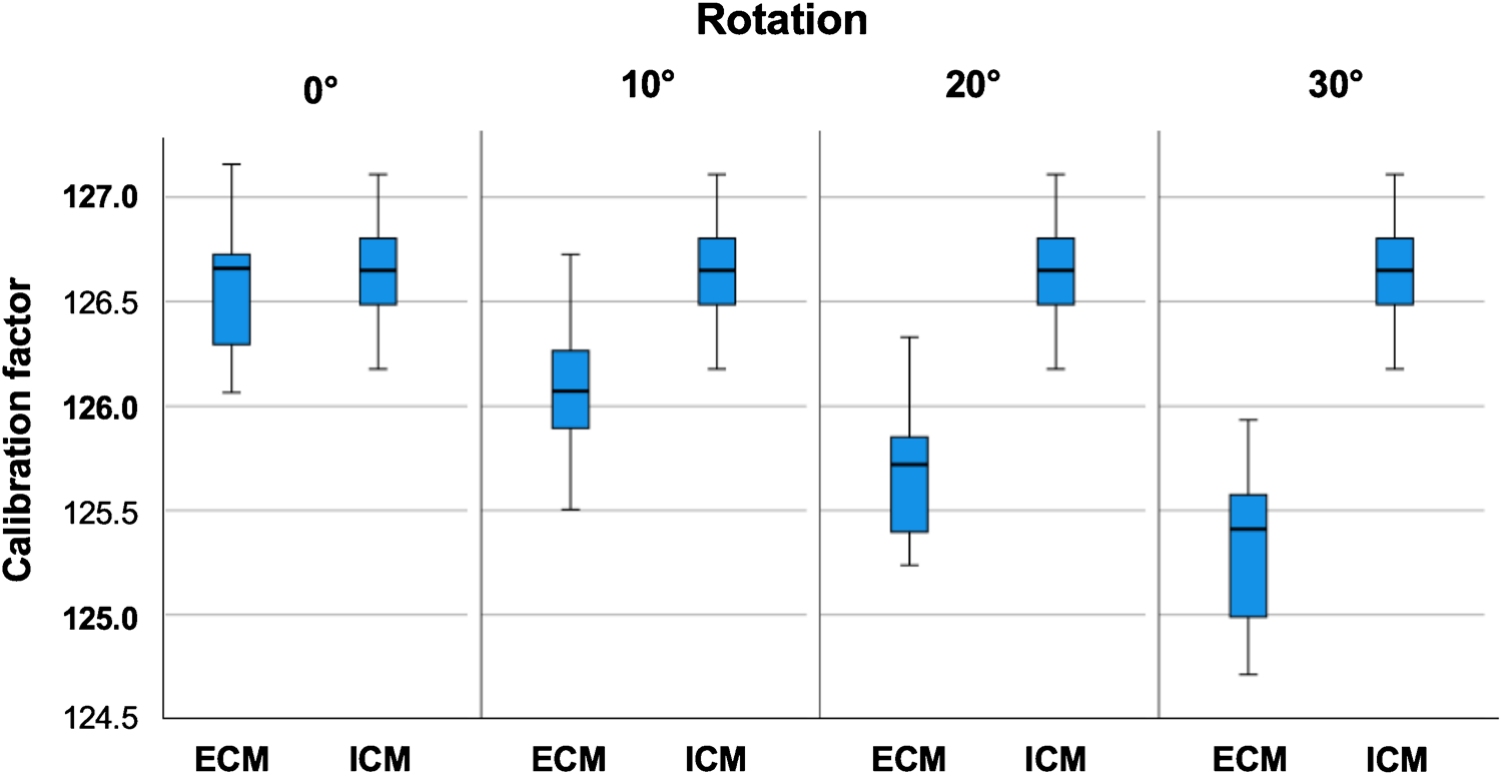
Box plots of ECM and ICM calibration factors by rotation. Note: the ICM is independent from rotation settings and therefore the same in all plots

The rotation-dependent calibration factors of ECM are plotted besides the rotation-independent calibration factors of the ICM in Fig. 7.

## Discussion

The primary objective of the presented study was the proof of concept of the bi-planar calibration method in a phantom model study. In twelve series with a total of 60 radiographs, the concept was established, and the method resulted in high reliability and validity. Overall, the difference between the calculated calibration factor of the ECM and the reference calibration factor (ICM) was below the clinical threshold of ≤ 1.5% in 93.8% of the cases. The difference was above 1.5% only in lateral radiographs with 30° rotation; and here only in on third of the cases.

While calibration of digital radiographs is the standard of care, there are significant limitations to the established methods [2, 4, 6]. The most common method with spherical markers between the legs at the supposed height of the hip joint above the detector is known to be highly unreliable with significant variance in quality and precision [2, 4, 6]. Errors have been reported to reach up to 28.6% and average errors being in the range of 5.2 to 12.5% in various independent studies [2, 4, 9, 14, 15]. More recently, dual-calibration methods with two markers as well as one marker have been introduced [8–10]. The dual-scale single-marker method uses a single marker in the same position as in the bi-planar method [9, 10]. However, the calculation of the calibration factor requires empirical data from CT scans or similar technologies [9, 10]. This method has resulted in a significant reduction of errors compared to conventional marker methods [9, 10]. Still, the errors were above the clinically relevant threshold in most cases.

The clinical threshold is based on a mathematical model and is dependent from magnification (calibration factor) as well as component size [12]. The effect of calibration errors increases proportional to magnification and component size. While the published steps were in 1% increments, a more detailed re-calculation showed the clinically relevant threshold to be ≤ 1.5% calibration error (Fig. 1S in Digital Supplement).

In conventional templating, the primary aim is implant selection and positioning, while correct sizing is often considered a secondary goal. Smith et al. reported that a precision of ± 1 implant size is widely accepted as sufficient [3]. Of note, this assumption is based on ex post analysis of achieved precision and therefore limited by the available methods. Additionally, even the established standard of ± 1 implant size is achieved in less than 75% of the reported cases and significant deviations are published. Thus, it can be summarized that current methods are neither precise nor reliable. To enable templating and sizing in particular to be integrated into robust patient pathways and potentially allow for integration of templating into computer-assisted surgery (e.g., robotic surgery), a reliable and robust method for calibration is required. Therefore, the threshold has been reduced to the subclinical value of ≤ 1.5% calibration error in the present study.

In the present phantom study, three different settings were considered: (a) anterior offset and (b) lateral offset of the marker and (c) rotation of the subject in the lateral radiograph. The anterior offset reflects patient’s soft tissue mass anterior to the pubic symphysis. A previous study on 400 CT scans has shown the distribution of the soft tissue in a wide range of subjects (Fig. 2S in Digital Supplement) [16]. Here, soft tissue thickness with a mean of 41 mm in a range of 14–117 mm (standard deviation: 16 mm) was found. Only 20 (5%) were above 79 mm. Therefore, it can be assumed that the settings of 20, 50, and 80 mm likely reflect the anatomy of the general population.

A lateral offset represents any deviation of the marker placement from the central beam which should be centered to the pubic symphysis. Deviation from the central beam would result in changed calculations that are corrected in the algorithms but may affect precision of the method. Therefore, settings of 0, 20, 40, and 60 mm were introduced to simulate possible clinical configurations. Deviations above 60 mm should be clearly identified as the marker would be far away from the laser marker prior to taking the radiograph.

Finally, rotation of the patient in lateral radiographs was considered a relevant variable and source of error as they are less common in clinical practice and the method may be impacted by rotation. As literature data on quality of lateral hip radiographs was not available, a wide range of error was assumed as possible with rotation of up to 30 degrees. Clinically, rotation of above 10° to 15° can be expected to be easily identifiable by radiology personnel while positioning the patient before taking the radiograph.

As shown in the phantom radiographs, lateral offset as well as rotation is easily identified in lateral radiographs. Additionally, anterior offset is identified in the lateral radiograph, but projection may be affected by both, lateral offset and rotation. The subgroup analysis showed that anterior offset as well as lateral offset had no relevant nonstatistically significant impact on the precision of the bi-planar calibration method. Rotation had a statistically significant impact but had only a limited effect of the actual difference of ECM and ICM calibration. A clinically relevant difference was only found on one-third of the cases with 30° rotation. Therefore, it can be concluded that rotation of up to 20° is acceptable, although strictly lateral radiographs should be aimed for. The underlying mathematical method was robust enough to counter rotational effects.

There are certain limitations to the method. First, this was a phantom study and proof of concept without real patients. A future clinical application is currently being planned. Here, additional factors influencing the method may be identified. Secondly, measurement methods may be improved. The measurements were performed by two independent reviewers after thorough training in the methodology. Here, reliability of most measurements was excellent with some cases of absolute agreement (so no ICC could be calculated due to lack of variance). The only variables with only mediocre reliability (with very small variation in absolute values) were related to the ICM. As the measurements can be automated, these variations may be reduced and further improve the precision of the method. Finally, the method requires object identification, measurements, and calculations which can be achieved by software either integrated into conventional templating software or dedicated standalone software. Manual calculations are not feasible in a clinical setting. Optimally, the process is not perceived by users of templating software and no additional actions are required during templating. Currently, this method is not yet integrated in available templating software.

## Conclusions

In conclusion, we have demonstrated that the bi-planar method precisely predicts the true calibration factor of the hip joint plane under various conditions. In lateral radiographs, rotation of up to 20° did not affect the quality and all images had calibration errors below the threshold for clinical significance.

## Supplementary Information

Below is the link to the electronic supplementary material.

Supplementary file1 (DOCX 277 KB)

## Data Availability

Supplemental content is submitted as part of the manuscript. No additional data and materials are available.
