# Supplementary material for: Bi-planar calibration method for templating of hip joint arthroplasty: phantom study and proof of concept
Source: Int Orthop. 2023 Mar 7;47(5):1249–57. doi: 10.1007/s00264-023-05747-4 (PMC10079716; doi:10.1007/s00264-023-05747-4)

Bi-planar calibration method for templating of hip joint arthroplasty: phantom-study and proof of concept

# Supplemental material

**Supplement 1. Table S1.** Intraclass correlation coefficients for inter-rater reliability.

| **Projection** | **Variable** | **Inter-rater reliability** | | | | | | **Descriptive statistics** | | | | **Descriptive statistics of paired measurements (Reviewer1 – Reviewer2)** | | |
| --- | --- | --- | --- | --- | --- | --- | --- | --- | --- | --- | --- | --- | --- | --- |
|  |  | **x-rays (n)** | **ICC** | **95% CI** | | | **p-value** | **Mean  in mm** | **min-max** | | **SD** | **Mean  in mm** | **min-max** | **SD** |
| Lateral | a | 48 | 1 | 1 | 1 | <0.001 | | 136.63 | 58.6 – 198.0 | 36.71 | | 0.19 | -1.0 – 2.0 | 0.64 |
|  | b | 48 | 0.992 | 0.982 | 0.996 | <0.001 | | 29.19 | 27.0 – 31.9 | 1.09 | | 0.09 | -0.2 – 1.1 | 0.18 |
|  | c | 48 | 1 | 1 | 1 | <0.001 | | 65.3 | 3.0 – 124.0 | 44.36 | | 0.15 | -0.9 – 1.1 | 0.45 |
|  | d | 48 | 1 | 1 | 1 | <0.001 | | 138.95 | 61.3 – 199.0 | 36.29 | | 0.02 | -2.0 – 3.0 | 0.87 |
| a.p. | h | 12 | 1 | 1 | 1 | <0.001 | | 52.47 | 17.9 – 99.7 | 28.47 | | -0.31 | -1.4 – 0.3 | 0.55 |
|  | i | 12 | 1 | 1 | 1 | <0.001 | | 46.15 | 0 – 98.9 | 33.77 | | -0.28 | -1.8 – 1-0 | 0.69 |
|  | k | 12 | 0.995 | 0.983 | 0.999 | <0.001 | | 28.77 | 27.7 – 29.9 | 0.86 | | 0 | -0.3 – 0.2 | 0.13 |
|  | m* | 12 | - | - | - | - | | 219.0 | -/- | 0 | | 0 | 0 – 0 | 0 |
|  | n | 12 | 0.483 | -0.471 | 0.841 | 0.118 | | 40.68 | 40.5 – 40.8 | 0.09 | | 0.05 | -0.1 – 0.2 | 0.1 |
|  | o | 12 | 0.859 | 0.537 | 0.959 | 0.001 | | 111.12 | 109.0 – 113.0 | 0.9 | | -0.25 | -1.0 – 1.0 | 0.6 |
|  | p | 12 | 0.656 | -0.081 | 0.898 | 0.038 | | 111.5 | 109.0 – 115.0 | 1.25 | | -0.50 | -3.0 – 1.0 | 1.2 |

*due to absolute agreement of all measurements there was no variance and therefore, ICCs could not be calculated.

Notes: Besides calculated ICCs, descriptive statistics for underlying variables are provided. These demonstrate means, ranges, and SD of variables as well as differences of paired measurements. The latter show the minimal differences without clinical significance.

**Supplement 2. Table S2.** Intraclass correlation coefficients for intra-rater reliability.

| **Projection** | **Variable** | **Intra-rater reliability** (25%) | | | | | **Descriptive statistics** | | | **Descriptive statistics of paired measurements (Delta variable) (R_1_-R_2_)** | | |
| --- | --- | --- | --- | --- | --- | --- | --- | --- | --- | --- | --- | --- |
|  |  | **x-rays (n)** | **ICC** | **95% CI** | | **p-value** | **mean**  **in mm** | **min-max** | **SD** | **mean**  **in mm** | **min-max** | **SD** |
| Lateral | a | 16 | 1 | 1 | 1 | <0.001 | 156.85 | 97.3 – 193.0 | 29.93 | -0.17 | -2.0 – 1.0 | 0.73 |
|  | b | 16 | 0.994 | 0.955 | 0.998 | <0.001 | 29.41 | 27.2 – 31.1 | 1.0 | 0.11 | -0.1 – 0.3 | 0.12 |
|  | c | 16 | 1 | 1 | 1 | <0.001 | 65.16 | 2.80 – 124.4 | 44.92 | 0.28 | -1.0 – 3.0 | 0.98 |
|  | d | 16 | 0.999 | 0.998 | 1 | <0.001 | 159.0 | 100.0 – 193.0 | 29.45 | 0 | -3.0 – 3.0 | 1.46 |
| a.p. | h | 4 | 1 | 0.997 | 1 | <0.001 | 53.84 | 19.4 – 90.8 | 27.04 | -0.5 | -1.0 – 0.1 | 0.47 |
|  | i | 4 | 1 | 0.998 | 1 | <0.001 | 47.21 | 3.7 – 89.4 | 32.03 | -0.52 | -1.8 – 0.1 | 0.86 |
|  | k | 4 | 0.991 | 0.882 | 0.999 | 0.002 | 29.31 | 28.7 – 29.9 | 0.53 | 0.03 | -0.1 – 0.2 | 0.13 |
|  | m* | 4 | - | - | - | - | 219.0 | -/- | 0 | 0 | 0 – 0 | 0 |
|  | n^ | 4 | - | - | - | - | 40.63 | 40.5 – 40.7 | 0.07 | 0 | -0.2 – 0.1 | 0.14 |
|  | o | 4 | 0.977 | 0.771 | 0.998 | 0.006 | 110.88 | 109.0 – 113.0 | 1.48 | -0.25 | -1.0 – 0 | 0.5 |
|  | p | 4 | 0.740 | -3.690 | 0.983 | 0.165 | 111.34 | 109.0 – 115.0 | 1.96 | -0.75 | -3.0 – 1.0 | 2.06 |

*due to absolute agreement of all measurements there was no variance and therefore, ICCs could not be calculated.

^ICC could not be calculated

Notes: Besides calculated ICCs, descriptive statistics for underlying variables are provided. These demonstrate means, ranges, and SD of variables as well as differences of paired measurements. The latter show the minimal differences without clinical significance including the variables without calculated ICCs.

Supplement 3. **Figure S1.** Table showing calculated deviations from optimal implant selection based on optimal implant size and calibration factor error. When calculations exceed 1.0 mm, the threshold for one size deviation is reached. These results are color-highlighted.
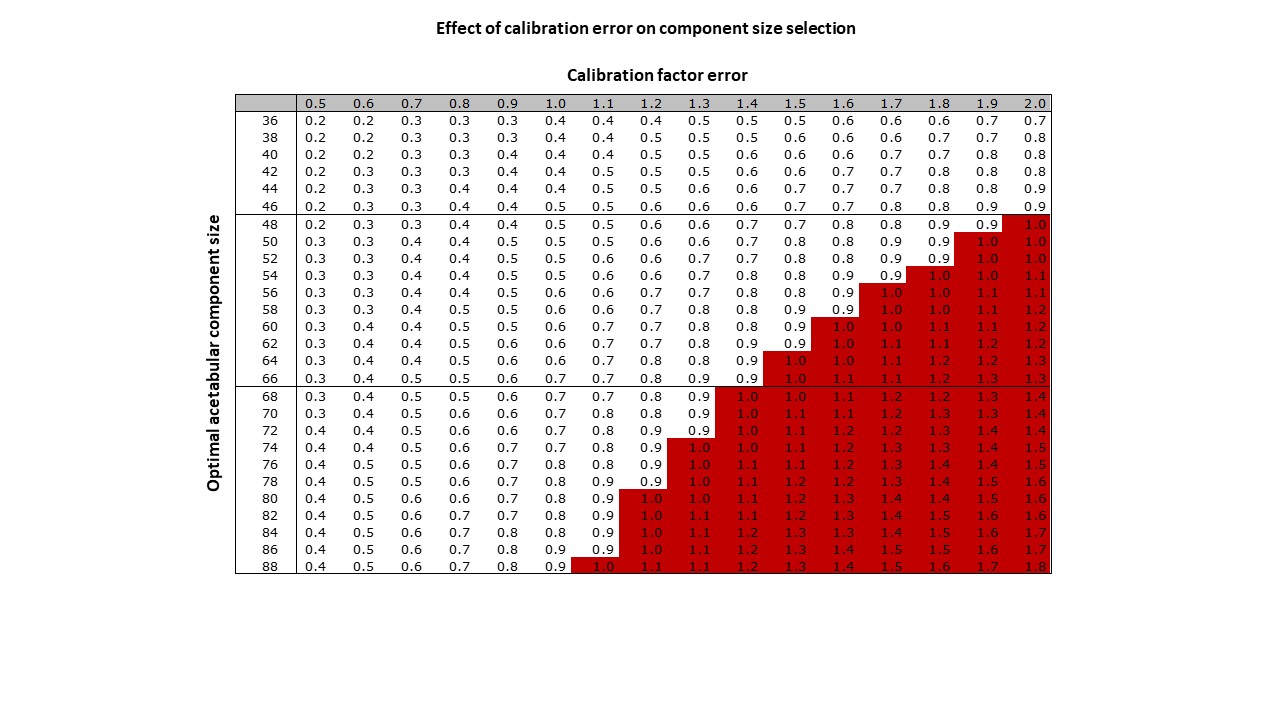


Supplement 4. **Figure S2.** Histogram of soft tissue anterior to the pubic symphysis from a cohort of 400 CT clinical scans.


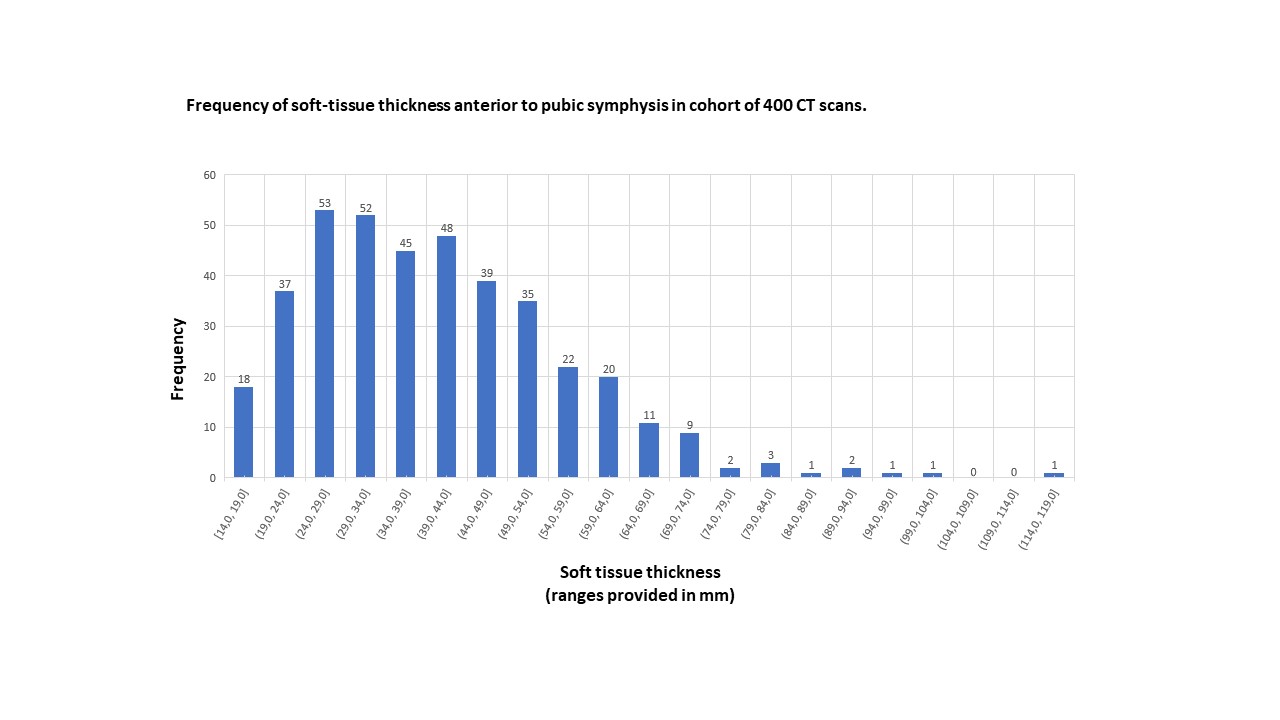

Supplement: Supplementary file 1 — Supplementary file1 (DOCX 277 KB) [file 264_2023_5747_MOESM1_ESM.docx]
